# *BMAL1* Suppresses Proliferation, Migration, and Invasion of U87MG Cells by Downregulating Cyclin B1, Phospho-AKT, and Metalloproteinase-9

**DOI:** 10.3390/ijms21072352

**Published:** 2020-03-28

**Authors:** Do hyeong Gwon, Woo-Yong Lee, Nara Shin, Song I Kim, Kuhee Jeong, Won-hyung Lee, Dong Woon Kim, Jinpyo Hong, Sun Yeul Lee

**Affiliations:** 1 Department of Medical Science, Chungnam National University School of Medicine, Daejeon 35015, Korea; dohyeong171@gmail.com (D.h.G.); s0870714@gmail.com (N.S.); kthddl2295@gmail.com (S.I.K.); visnu528@cnu.ac.kr (D.W.K.); 2 Department of Anatomy, Brain Research Institute, Chungnam National University School of Medicine, Daejeon 35015, Korea; 3 Department of Orthopedic Surgery, Regional Rheumatoid and Degenerative Arthritis Center, Chungnam National University Hospital, Chungnam National University School of Medicine, Daejeon 35015, Korea; studymachine@hanmail.net; 4 Department of Anesthesia and Pain Medicine, Chungnam National University Hospital, Chungnam National University School of Medicine, Daejeon 35015, Korea; kuheeinkorea@gmail.com (K.J.); whlee@cnu.ac.kr (W.-h.L.); 5 Department of Neuroscience and Physiology, and Dental Research Institute, School of Dentistry, Seoul National University, Seoul 08826, Korea

**Keywords:** glioblastoma, BMAL1, proliferation, migration, invasion

## Abstract

Several studies have shown that brain and muscle aryl hydrocarbon receptor nuclear translocator-like 1 (BMAL1), an important molecule for maintaining circadian rhythms, inhibits the growth and metastasis of tumor cells in several types of cancer, including lung, colon, and breast cancer. However, its role in glioblastoma has not yet been established. Here, we addressed the function of BMAL1 in U87MG glioblastoma cells with two approaches—loss and gain of function. In the loss of function experiments, cell proliferation in U87MG cells transfected with small interfering RNA (siRNA) targeting BMAL1 was increased by approximately 24% (small interfering (si)-NC 0.91 ± 0.00 vs. si-BMAL1 1.129 ± 0.08) via upregulation of cyclin B1. In addition, cell migration and invasion of BMAL1 siRNA-treated glioblastoma cells were elevated by approximately 20% (si-NC 51.00 ± 1.53 vs. si-BMAL161.33 ± 0.88) and 209% (si-NC 21.28 ± 1.37 vs. si-BMAL1 44.47 ± 3.48), respectively, through the accumulation of phosphorylated-AKT (p-AKT) and matrix metalloproteinase (MMP)-9. Gain of function experiments revealed that adenovirus-mediated ectopic expression of BMAL1 in U87MG cells resulted in a 19% (Adenovirus (Ad)-vector 0.94± 0.03 vs. Ad-BMAL1 0.76 ± 0.03) decrease in cell proliferation compared with the control via downregulation of cyclin B1 and increased early and late apoptosis due to changes in the levels of BCL2-associated X protein (BAX), B-cell lymphoma 2 (BCL-2), and cleaved caspase-3. Likewise, cell migration and invasion were attenuated by approximately 24% (Ad-vector 55.00 ± 0.00 vs. Ad-BMAL1 41.83 ± 2.90) and 49% (Ad-vector 70.01 ± 1.24 vs. Ad-BMAL1 35.55 ± 1.78), respectively, in BMAL1-overexpressing U87MG cells following downregulation of p-AKT and MMP-9. Taken together, our results suggest that BMAL1 acts as an anti-cancer gene by altering the proliferation, migration, and invasion of glioblastoma cells. Therefore, the BMAL1 gene could be a potential therapeutic target in the treatment of glioblastoma.

## 1. Introduction

Glioblastoma is the most malignant type of tumor in the central nervous system, largely originating from astrocytes, and occurs at a rate of 3 per 100,000 people per year [1]. The median survival time following diagnosis of glioblastoma is approximately 12 to 15 months, and only 3%–5% of patients live for more than 5 years when treated with surgery, radiotherapy, and chemotherapy [1]. Until now, there has only been one drug approved by the Food and Drug Administration for the treatment of glioblastoma: temozolomide (TMZ). TMZ alkylates or methylates the N-7 or O-6 positions, respectively, of guanine residues in DNA and triggers tumor cell death, and this drug is favored for the treatment of malignant brain tumors such as astrocytoma and glioblastoma. TMZ also provides an anti-tumor effect by inducing the damage-associated molecular patterns from glioblastoma and enhancing the tumor-specific immune responses [2]. TMZ, however, has several side effects, including bone marrow suppression, nausea, vomiting, inhibition of blood vessel growth, coronary artery disease, and peripheral artery disease [3]. In addition, glioblastoma is occasionally insensitive to TMZ as some tumor cells express O6-alkylguanine DNA alkyltransferase, which allows repair of this type of DNA damage [4]. Therefore, the development of new innovative medicine for the treatment of glioblastoma with less toxicity and high efficacy is urgently required.

The circadian rhythm is the hormonal time-keeping system that establishes the daily rhythm of biological, biochemical, physiological, and behavioral function in animals, plants, and bacteria [5]. In mammals, circadian rhythm genes are regulated by a negative feedback loop [6]. Brain and muscle aryl hydrocarbon receptor nuclear translocator-like 1 (BMAL1)-Clock heterodimers bind to E-box elements in the promoters of the Period (PER) and Cryptochrome (CRY) genes, activating transcription. Then, PER–CRY complexes interact with BMAL1–CLOCK complexes, repressing their activities. Several lines of evidence have indicated that the dysregulation of circadian rhythms is tightly linked with several pathologic conditions, including cancer, diabetes, neurodegenerative disease, aging, oxidative stress, and depression [7,8,9,10,11,12]. In particular, the disruption of circadian rhythms is closely associated with the development and progression of various forms of human cancers, including breast, prostate, and lung cancer [13]. For instance, the mRNA levels of Per1 and Per2 are reduced in sporadic and familial breast tumors compared with normal breast tissue [14]. Per1 expression in prostate cancer is also significantly lower compared with normal prostate tissue [15].

It has been reported that BMAL1, a key transcription factor in circadian rhythms, regulates the incidence and maintenance of tumor cells in several types of cancer, such as ovarian cancer, lymphocytic leukemia, and prostate cancer [16,17,18]. For example, BMAL1 inhibits entry into S-phase of the cell cycle in human colon cancer [19] and suppresses cell invasion in A549 lung cancer cells by inhibiting the phosphoinositide 3-kinase-Akt-matrix metalloproteinase (MMP)-2 signaling pathway [20]. By contrast, BMAL1 depletion inhibits the cell cycle in malignant pleural mesothelioma with the downregulation of Wee1, cyclin B, and p21 and the upregulation of cyclin E [21]. However, the mechanism of BMAL1 in glioblastoma has not yet been elucidated.

In this study, we addressed the role of BMAL1 in glioblastoma cells in vitro with two basic approaches, loss and gain of function. We tested whether BMAL1 could influence proliferation, migration, and invasion of BMAL1-silenced or BMAL1-overexpressing glioblastoma cells with the aid of several assays. Our results suggest that BMAL1 may function as an anti-glioblastoma gene in cell growth, migration, and invasion by regulating Cyclin B, phosphorylated -AKT (p-AKT), and metalloproteinase (MMP)-9 signaling pathways.

## 2. Results

### 2.1. BMAL1 Was Effectively Silenced in BMAL1 SiRNA-Treated U87MG Glioblastoma Cells

Prior to investigating the role of BMAL1 in glioblastoma cells with an RNA interference (RNAi) approach, we validated whether our BMAL1 small interfering RNA (siRNA)could silence the BMAL1 gene specifically in glioblastoma cells.

**Figure 1:**
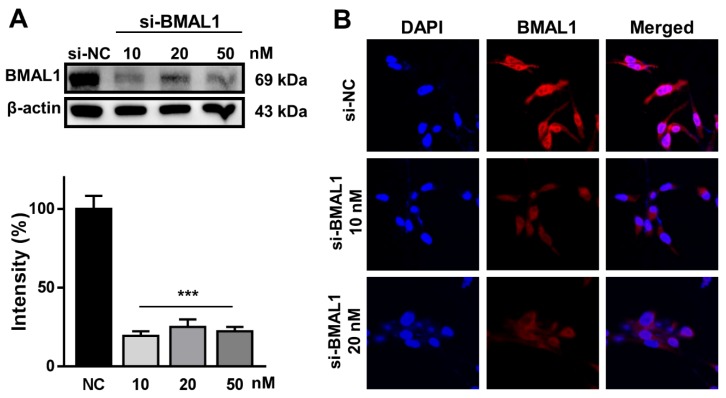
Brain and muscle aryl hydrocarbon receptor nuclear translocator-like 1 (BMAL1) is effectively silenced in BMAL1 small interfering RNA (siRNA)-treated U87MG cells. (**A**) U87MG cells were incubated with negative control siRNA (si-NC; 10 nM) or BMAL1 siRNA (si-BMAL1; 10, 20, and 50 nM). After 48 h, the protein level of BMAL1 was examined by Western blot with anti-BMAL1 antibodies, normalized with β-actin, and calculated as a percentage of the si-NC. Data are expressed as the mean ± standard error of the mean (SEM) (one-way ANOVA test, *** *p* < 0.001 vs. si-NC, three independent experiments). (**B**) Immunofluorescence of BMAL1 expression in U87MG cells transfected with si-NC (10 nM) or si-BMAL1 (10 and 20 nM).

To examine this, U87MG glioblastoma cells were treated with negative control (small interfering (si)-NC; 10 nM) or BMAL1 (si-BMAL1; 10, 20, and 50 nM) siRNA for 2 days. Western blot analysis using anti-BMAL1 antibodies revealed that the level of BMAL1 protein in BMAL1 siRNA-treated cells was decreased by approximately 76% compared with NC siRNA-treated cells (si-NC59.03 ± 4.47 vs.si-BMAL1 14.05 ± 2.91) (Figure 1A). Similarly, the reduction of BMAL1 in cells treated with BMAL1 siRNA was observed by immunofluorescent staining with anti-BMAL1 antibodies (Figure 1B).

These data suggested that this BMAL1 siRNA specifically knocked down the BMAL1 protein in U87MG cells.

### 2.2. BMAL1 Silencing Promoted the Proliferation of U87MG Cells

**Figure 2:**
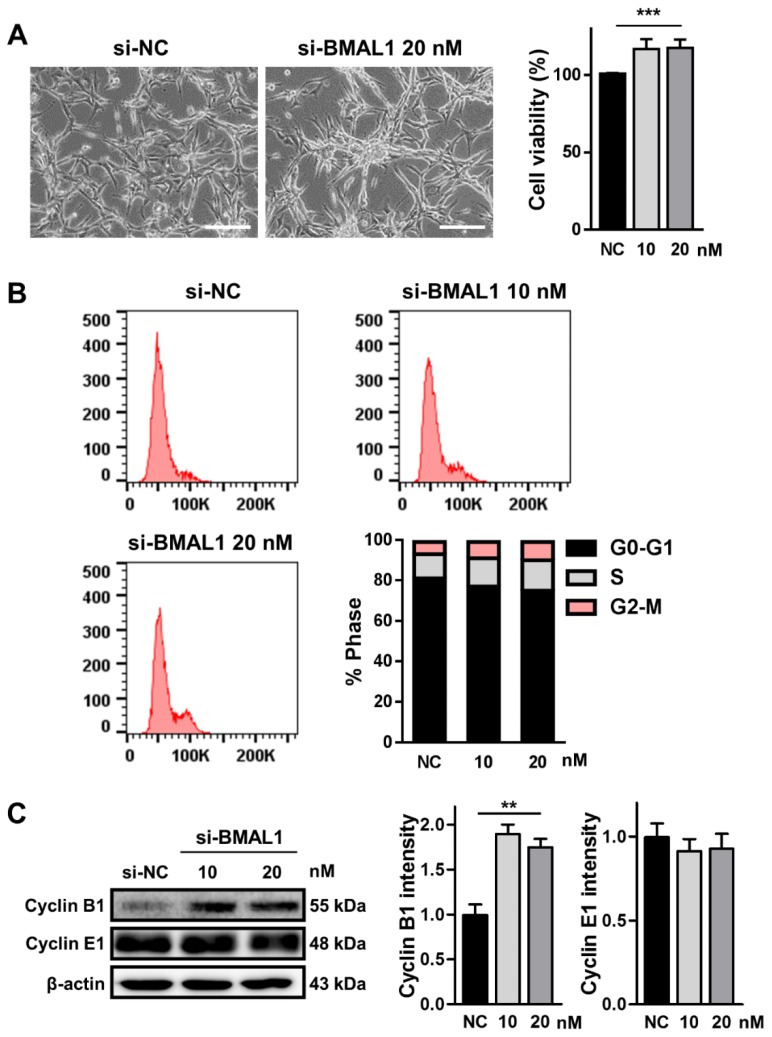
Knockdown of BMAL1 in U87MG cells by BMAL1 siRNA increased cell proliferation via upregulation of cyclin B1. (**A**) U87MG cells were incubated with si-NC (10 nM) and si-BMAL1 (10 and 20 nM). Scale bar: 50 μm. The proliferation was assessed by 3-(4,5-dimethylthiazol-2-yl)-2,5-diphenyltetrazolium bromide (MTT) assay at 48 h post-transfection. Data are expressed as the mean ± SEM (one-way ANOVA test, *** *p* < 0.001 vs. NC, three independent experiments). (**B**) The DNA contents of cells treated with si-NC and si-BMAL1 in each stage of the cell cycle was analyzed by flow cytometry from three independent experiments. (**C**)The expression levels of cyclin B1 and cyclin E1 in cells treated with si-NC and si BMAL1 were examined by Western blot. Data are expressed as the mean ± SEM (one-way ANOVA test, ** *p* < 0.01 vs. NC, three independent experiments).

Several reports have suggested that BMAL1 can suppress the growth of tumor cells in some cancers, such as prostate cancer, epithelial ovarian cancer, and lymphocytic leukemia [16,17,18]. Hence, we performed an3-(4,5-dimethylthiazol-2-yl)-2,5-diphenyltetrazolium bromide (MTT)assay to investigate whether BMAL1 knockdown could increase the division of glioblastoma cells. When U87MG cells were treated with BMAL1 siRNA (10 and 20 nM) for 2 days, cell number was increased by approximately 24% compared with the NC siRNA-treated control cells (si-NC 0.91± 0.008 vs. si-BMAL1 1.129 ± 0.08) as determined by the MTT assay (Figure 2A). To further elucidate the mechanism underlying the increased proliferation of these cells, we analyzed the cell cycle in BMAL1 siRNA-treated cells using flow cytometry. The number of propidium iodide (PI)-stained cells in G2/M phase was increased by approximately 45% and 65%, respectively, in BMAL1 siRNA (10 and 20 nM)-treated cells compared with the NC siRNA-treated cells (si-NC10 nM 5.49 ± 0.19 vs.si-BMAL1 10 nM 7.96 ± 0.86 vs. si-BMAL1 20 nM 9.04 ± 1.30; one-way ANOVA test with Dunnett’s post-hoc test, *F*(2, 9) = 5.395, *p* = 0.0288, * *p* < 0.05 (si-NC vs. si-BMAL1 20 nM)) (Figure 2B). Furthermore, we examined the level of cyclin B1 and cyclin E1, two key molecules in the G2/M transition of the cell cycle [21]. Western blot analysis revealed that the level of cyclin B1 protein in U87MG cells incubated with BMAL1 siRNA (10 and 20 nM) was increased by approximately 90% with cells incubated with NC siRNA, whereas that of cyclin E1 did not change (si-NC 26.48 ± 3.19 vs. si-BMAL1 50.27 ± 2.90) (Figure 2C).

Our results suggest that the accumulation of cyclin B1 protein might contributed to the increased growth of BMAL1 siRNA-transfected U87MG cells.

### 2.3. BMAL1 Silencing Increased the Cell Migration and Invasion in BMAL1 SiRNA-Treated U87MG Cells

**Figure 3:**
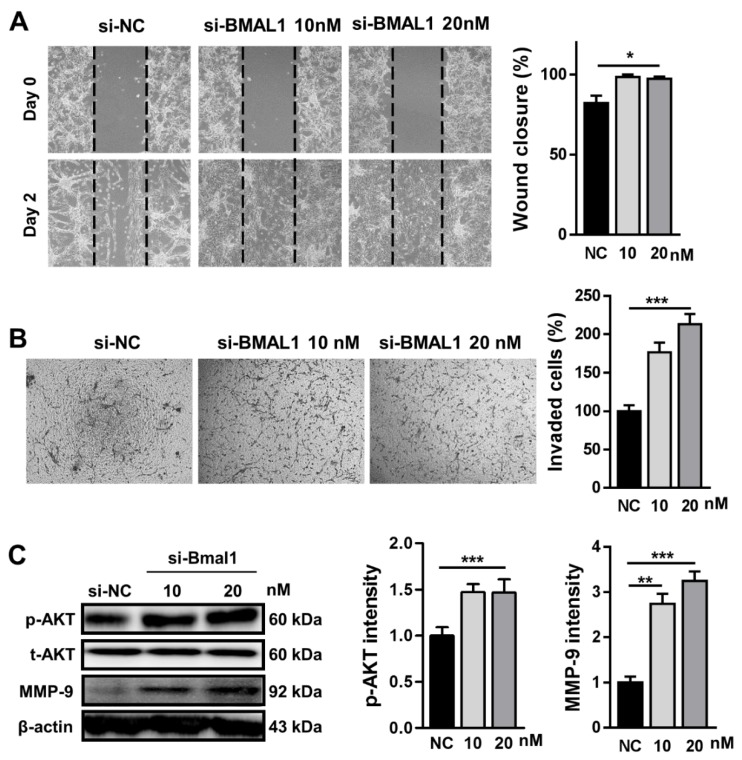
Knockdown of BMAL1 increased cell migration and invasion by upregulation of phosphorylated AKT (p-AKT) and matrix metalloproteinase (MMP)-9 in BMAL1 siRNA-transfected U87MG cells. (**A**) Cell migration was analyzed by a wound healing assay in si-NC and si-BMAL1 siRNA-treated U87MG cells at 48 h after scratching. Data are expressed as the mean ± SEM (one-way ANOVA test, * *p* < 0.05 vs. NC, three independent experiments). (**B**) Invasion of si-NC-and si-BMAL1-treated cells into the matrix were analyzed. Data are expressed as the mean ± SEM (one-way ANOVA test, *** *p* < 0.001 vs. NC, three independent experiments). (**C**) Expression levels of p-AKT and MMP-9 were analyzed by Western blot in si-NC- andsi-BMAL1-treated U87MG cells. Data are expressed as the mean ± SEM (one-way ANOVA test, ** *p* < 0.01 and *** *p* < 0.001 vs. NC, three independent experiments).

Next, we investigated whether BMAL1 silencing could influence cell migration using a wound healing assay. Migration of BMAL1 siRNA-transfected U87MG cells was increased by approximately 20% compared with NC siRNA-transfected cells (si-NC51.0 ± 1.53 vs.si-BMAL1 61.33 ± 0.88) (Figure 3A). Consistent with previous reports that p-AKT regulates cell migration, the level of p-AKT was increased by approximately 43% in BMAL1 siRNA-treated cells compared with NC siRNA-treated cells (si-NC 33.61 ± 3.12 vs.si-BMAL1 48.12 ± 7.15) (Figure 3C).

We also evaluated whether BMAL1 has a role in the invasion of U87MG cells in vitro. In the Matrigel invasion assay, the number of invaded cells was increased by approximately 208% in BMAL1-silenced U87MG cells compared with control cells (si-NC 21.28 ± 1.37 vs.si-BMAL1 44.47 ± 3.48) (Figure 3B). Then, to test the possibility that BMAL1 could promote cell invasion via upregulation of p-AKT and MMP-9, we investigated the levels of p-AKT and MMP-9 in BMAL1-silenced cells by Western blot. p-AKT and MMP-9 levels were increased by approximately 43% and 325%, respectively, compared with NC siRNA-treated cells (p-AKT:si-NC33.61± 3.12 vs.si-BMAL1 48.12 ± 7.1; MMP-9: si-NC13.89 ± 1.82 vs.si-BMAL1 45.18 ± 2.88) (Figure 3C).

Collectively, the increase of cell migration and invasion in BMAL1-silenced U87MG cells might be related with the upregulation of p-AKT and/or MMP-9.

### 2.4. BMAL1 Expression Increasedin Ad-BMAL1 Virus-Infected U87MG Cells

**Figure 4:**
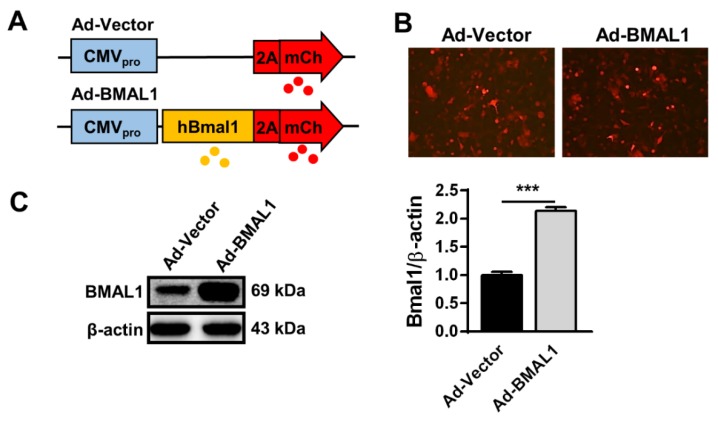
BMAL1 was highly expressed in U87MG cells infected with Ad-BMAL1 viruses. (**A**) Construction of Ad-vector (control) and Ad-BMAL1 (BMAL1) plasmids. (**B**) U87MG cells were infected with control and BMAL1 viruses for 48 h. Expression of mCherry was examined under the fluorescence microscope. (**C**) The cell lysates from Figure 5B were also analyzed by Western blot to determine the protein level of BMAL1 by viral infection. Data are expressed as the mean ± SEM (Student’s *t*-test, *** *p* < 0.001 vs. Ad-vector, three independent experiments).

We revealed above that BMAL1 knockdown accelerates cell growth, migration, and invasion of U87MG cells. Thus, to confirm the function of BMAL1 in glioblastoma with gain of function experiments, we prepared Adenovirus-vector (Ad-Vector) and Adenovirus-BMAL1 (Ad-BMAL1) constructs (Figure 4A) and then produced high-titer adenoviruses (~1 × 10^9^ pfu/mL) in HEK293A cells (see Materials and Methods section). Then, we evaluated the integrity of the prepared viruses in two ways. Following infection of U87MG cells with control or BMAL1 adenovirus for 2 days, mCherry expression was observed under a fluorescence microscope (Figure 4B), which revealed that BMAL1 expression was increased by approximately 91% in Ad-BMAL1 virus-infected U87MG cells compared with control virus-infected cells (Ad-vector 44.12 ± 6.10 vs. Ad-BMAL1 84.19 ± 3.02) (Figure 4C).

Therefore, we used these viruses in further experiments to reveal the role of BMAL1 in glioblastoma.

### 2.5. Cell Proliferation Decreases in Ad-BMAL1 Virus-Infected U87MG Cells

**Figure 5:**
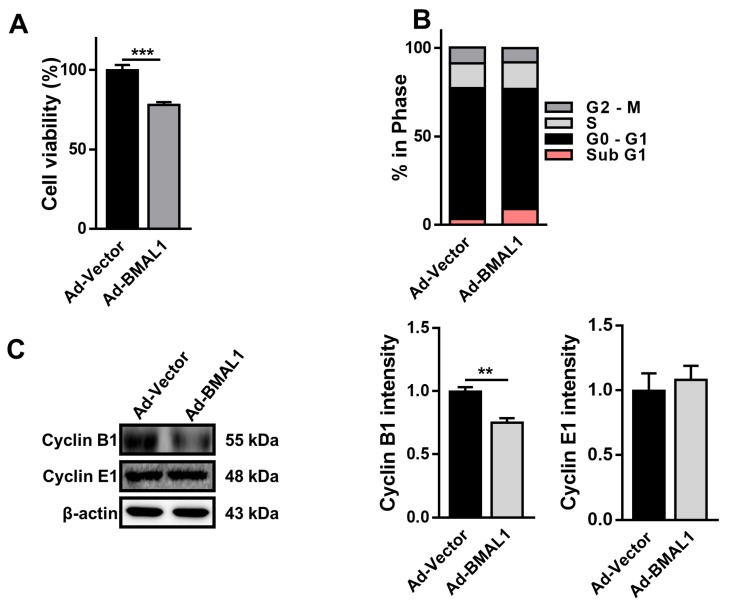
Cell proliferation was decreased in Ad-BMAL1 virus-infected U87MG cells via downregulation of cyclin B1 and increased apoptosis. (**A**) U87MG cells were infected with control and BMAL1 adenovirus for 48 h, and cell viability was measured using MTT assay. Data are expressed as the mean ± SEM (Student’s *t*-test, *** *p* < 0.001 vs. Ad-vector, three independent experiments). (**B**) Cell cycle analysis was performed as described in Figure 2B. These are representative data from three independent experiments. (**C**) Lysates from control- and BMAL1 virus-infected U87MG cells were used to determine the level of cyclin B1 and cyclin E1 by Western blotting. Data are expressed as the mean ± SEM (Student’s *t*-test, ** *p* < 0.01 vs. Ad-vector, three independent experiments).

To explore whether ectopic expression of BMAL1 may influence the proliferation of glioblastoma cells, U87MG cells were infected with control or BMAL1 viruses for 2 days and MTT assay was performed. The viability of Ad-BMAL1 virus-infected cells was decreased by approximately 22% compared with control virus-infected cells (Ad-vector 0.94 ± 0.03 vs. Ad-BMAL1 0.73 ± 0.03) (Figure 5A). Analysis of the cell cycle using flow cytometry revealed that the number of sub-G1 cells in the Ad-BMAL1 virus-infected cell population was increased by approximately 255% compared with control virus-infected cells (Ad-vector 3.38 ± 0.21 vs. Ad-BMAL1 8.61 ± 1.10) (Figure 5B).

Because BMAL1 knockdown in glioblastoma cells upregulates cyclin B1 expression (Figure 2C), BMAL1 overexpression in cells might downregulate cyclin B1 expression. As expected, Western blot analysis revealed that the protein level of Cyclin B1 in Ad-BMAL1 virus-infected U87MG cells was decreased by approximately 22% compared with control virus-infected cells (Ad-vector 71.69 ± 5.95 vs. Ad-BMAL1 55.93 ± 4.82) (Figure 5C).

**Figure 6:**
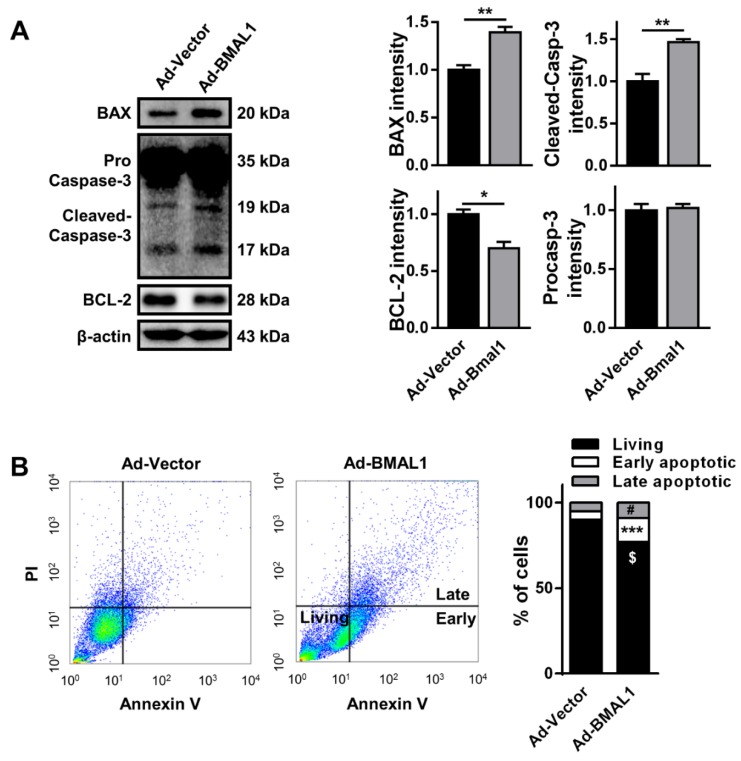
Apoptosis increases in Ad-BMAL1 virus-infected U87MG cells. (**A**)The expression levels of BAX, pro-caspase-3, cleaved-caspase-3 (both 19 kDa and 17 kDa), and BCL-2 were measured with ImageJ, normalized with β-actin, and then expressed as a percentage of Ad-vector by Western blot. Data are expressed as the mean ± SEM (Student’s t-test, * *p* < 0.05 and ** *p* < 0.01 vs. Ad-vector, three independent experiments). (**B**) U87MG cells treated with control and BMAL1adenoviruses were stained with annexin-V and propidium iodide (PI); the percentage of living, early (annexin-V positive), and late (annexin-V/PI positive) apoptotic cells were investigated by flow cytometry. These are representative data from three independent experiments. Data are expressed as the mean ± SEM (unpaired Student’s *t*-test; ^$^ *p* < 0.01 vs. living cells of Ad-BMAL1, *** *p* < 0.001 vs. early apoptotic cells of Ad-BMAL1, ^#^ *p* < 0.05 vs. late apoptotic cells of Ad-BMAL1; three independent experiments).

In addition, the reduction in cell viability could have been caused by an increased rate of apoptosis, which could be a result of changes in the levels of apoptotic proteins [22]. Hence, we analyzed the expression of the apoptosis-related proteins BCL2-associated X protein (BAX), caspase-3, and B-cell lymphoma 2 (BCL-2). Interestingly, the levels of the pro-apoptotic molecules BAX and cleaved caspase-3 were increased, respectively, by approximately 50% and 98%(BAX: Ad-vector 51.67 ± 4.06vs.Ad-BMAL177.26 ± 3.0; cleaved caspase-3: Ad-vector 36.18 ± 3.16 vs. Ad-BMAL1 71.61 ± 1.59),whereas that of the anti-apoptotic molecule BCL-2 was decreased by approximately 27% in Ad-BMAL1 virus-infected cells compared with control virus-infected cells(Ad-vector 81.88 ± 3.37 vs.Ad-BMAL1 59.80 ± 2.44) (Figure 6A). In another analysis with annexin V/PI staining to detect early and late apoptotic cells, the number of early (annexin V-positive) and late (annexin V/PI-positive) apoptotic cells were also significantly increased respectively by approximately 285% (Ad-vector 4.97 ± 0.20 vs. Ad-BMAL1 14.18 ± 0.61; unpaired Student’s *t*-test, *** *p* < 0.001 vs. Ad-vector) and 179% (* *p* < 0.05; Ad-vector 4.97 ± 1.06 vs. Ad-BMAL1 8.91 ± 0.84; unpaired Student’s *t*-test, * *p* < 0.05 vs Ad-vector), whereas that of living cells was significantly decreased by approximately 15% (Ad-vector 90.06 ± 1.20 vs. Ad-BMAL1 76.91 ± 1.39; unpaired Student’s *t*-test, ** *p* < 0.01 vs Ad-vector) in Ad-BMAL1 virus-infected cells compared with control-virus infected cells (Figure 6B).

Collectively, these data suggested that the reduction of proliferation in BMAL1-overexpressing U87MG cells might be related with the downregulation of cyclin B1 and the increase of early and late apoptotic cells.

### 2.6. Cell Migration and Invasion Decreasedin BMAL1 Overexpression in U87MG Cells

**Figure 7:**
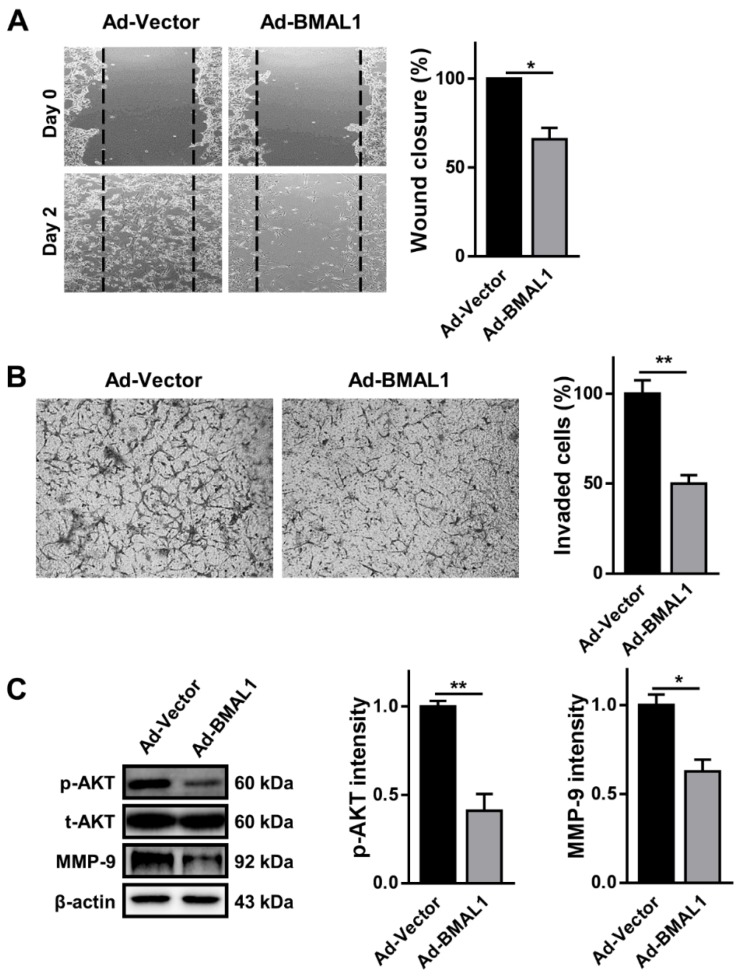
Cell migration and invasion are decreased in U87MG cells with overexpression of BMAL1. (**A**) At 48 h post-infection with control or BMAL1 adenovirus in U87MG cells, the near-confluent cell culture was scratched with a 20 µl tip at the center of wells. Migration was expressed as a percentage of wound closure at 48 h after scratching. Data are expressed as the mean ± SEM (one-way ANOVA test, * *p* < 0.05 vs. NC, three independent experiments). (**B**) Invaded cells infected with control and BMAL1 viruses were analyzed. Data are expressed as the mean ± SEM (Student’s *t*-test, ** *p* < 0.01 vs. Ad-vector, three independent experiments). (**C**) Expression levels of p-AKT and MMP-9 in U87MG cells infected with control and BMAL1 viruses were examined by Western blot. Data are expressed as the mean ± SEM (Student’s *t*-test, * *p* < 0.05 and ** *p* < 0.01 vs. Ad-vector).

With the same rationale as for cell proliferation in glioblastoma cells above, we examined the role of BMAL1 in the migration and invasion of glioblastoma cells with a gain of function approach. In the wound healing assay, cell migration was decreased by approximately 30% in U87MG cells infected with BMAL1 viruses compared with control virus-infected cells (Ad-vector 55.00 ± 0.00 vs. Ad-BMAL1 38.83 ± 2.89) (Figure 7A). In contrast with the silencing data (Figure 3C), the level of p-AKT was decreased by approximately 55% in BMAL1 virus-treated cells compared with the negative control (Ad-vector 82.83 ± 2.65 vs. Ad-BMAL1 37.06 ± 7.75) (Figure 7C).

In addition, we carried out the Matrigel invasion assay using control or BMAL1 virus-infected U87MG cells to measure invasiveness. The number of cells that penetrated the pores of the membrane was decreased by approximately 50% in BMAL1-overexpressing U87MG cells compared with control cells (Ad-vector 70.01 ± 1.23 vs. Ad-BMAL1 35.55 ± 1.77) (Figure 7B). Similarly, the levels of MMP-9 in BMAL1-overexpressing cells decreased by approximately 33%, compared with control cells (Ad-vector 75.17 ± 4.43 vs. Ad-BMAL1 50.44 ± 7.58) (Figure 7C).

Altogether, the loss of migration and invasion in ectopic BMAL1-expressing U87MG cells might be related with the downregulation of p-AKT and MMP-9.

## 3. Discussion

In this study, we sought to determine whether an uncontrolled circadian rhythm due to abnormal expression of BMAL1, a key regulator of the circadian clock, could influence on the tumor-promoting activities of glioblastoma cells. siRNA-dependent BMAL1 silencing in U87MG glioblastoma cells accelerated cell proliferation, migration, and invasion. On the other hand, adenovirus-mediated ectopic expression of BMAL1 in glioblastoma cells resulted in the inhibition of cell proliferation, migration, and invasion.

Our results are consistent with previous studies [21,23,24]. The core circadian clock gene BMAL1 acts as a potential anti-oncogene in pancreatic cancer [25]; the loss of BMAL1 with PER2 accelerates lung tumorigenesis [26], and BMAL1 inhibits tumorigenesis and further increases the sensitivity of tongue squamous cell carcinoma to paclitaxel [24]. These data suggest the decreased expression of BMAL1 in different types of cancer may disrupt the cell cycle, possibly initiating and accelerating cancer progression. However, there is also some conflicting evidence—BMAL1 levels are elevated in malignant pleural mesothelioma (MPM) [21] and hepatologic malignancies [16]. Thus, BMAL1 depletion in MPM cells suppressed cell proliferation due to disruption of the cell cycle with a substantial increase in apoptotic and polyploid cell populations [21]. Altogether, these results indicated that BMAL1 may promote or inhibit oncogenesis depending on the types of cancer.

Dysregulation of cell cycle regulators, including cyclin B, could impair cell proliferation due to disruption of the cell cycle and consequent apoptotic cell death [21]. For instance, the loss of cyclin B below a critical basal level in H1299 lung carcinoma cells results in cell cycle disruption and apoptosis [27]. Likewise, we observed that the level of cyclin B was downregulated in BMAL1-overexpressing glioblastoma cells, which likely caused the increase in the sub-G1 population by apoptosis [21]. Immunoblot analysis revealed that the pro-apoptotic markers BAX and cleaved caspase-3 were increased and the anti-apoptotic marker BCL-2 was decreased in BMAL1-overexpressing glioblastoma cells compared with control cells (Figure 5). These data imply that ectopic expression of BMAL1 in glioblastoma cells may result in the decrease of cyclin B expression, thereby contributing to the increase in the number of sub-G1 cells, which are a result of apoptotic cell death. In addition, several lines of evidence have shown that BMAL1 decreases the migration and invasion of tumor cells in several types of cancer, including tongue squamous cell carcinoma and lung cancer [20,24]. To illustrate, in A549 lung cancer cells, BMAL1 knockdown stimulates cancer cell invasion, whereas BMAL1 overexpression lowers cellular invasion [20]. In a similar manner, when BMAL1 was ectopically expressed via adenovirus in U87MG cells, the migration and invasion of cells declined in the wound healing and Matrigel assays through the downregulation of p-AKT and MMP-9 signaling pathways, which are known to coordinate the migration and invasion of tumor cells (Figure 6). In addition, dichloroacetate, a pyruvate dehydrogenase kinase 1 inhibitor, together with radiotherapy can effectively sensitize glioblastoma cells by inducing the cell-cycle arrest at the G2/M phase [28]. Thus, in a further study, we will investigate whether BMAL1 with irradiation could further suppress tumorigenesis of glioblastoma cells.

Furthermore, several reports have indicated that BMAL1 could increase the sensitivity of malignant tumor cells to chemotherapeutics [21,23,24]. For example, BMAL1 silencing lowers the efficacy of dexamethasone on growth of B16 melanoma cancer cells [29]. BMAL1 also increases sensitivity to paclitaxel in tongue squamous cell carcinoma by recruiting enhancer of zeste homolog 2 (EZH2) repressors to the telomerase reverse transcriptase (TERT) promoter to prohibit TERT transcription [24]. Overexpression of BMAL1 has also been shown to increase the responsiveness of colorectal cancer to oxaliplatin [23]. Interestingly, cell viability was significantly decreased in U87MG cells co-treated with BMAL1 viruses and TMZ compared to cells treated with BMAL1 adenovirus or TMZ alone (data not shown). Although we should carry out additional experiments as a further study to prove the exact role of BMAL1 in this condition, it is likely that ectopic BMAL1 expression may enhance the responsiveness of U87MG cells to chemotherapeutics by increasing the phosphorylation of a histone H2A variant (H2AX) and the activation of apoptosis [30].

## 4. Materials and Methods

### 4.1. Cell Culture

The human glioblastoma cell line U87MG was purchased from the Korean Cell Line Bank (KCLB, Seoul, Korea) and maintained in minimum essential medium (Welgene Biotech, Kyungsan, Kyeongbuk, Korea) supplemented with 10% heat-inactivated fetal bovine serum (FBS) and 1% antibiotics in a humidified 5% CO_2_ incubator at 37 °C. All cell experiments were carried out at the same time point (10 AM) to avoid circadian variation by BMAL1 expression.

### 4.2. Small Interfering RNA (SiRNA) Transfection

U87MG cells were plated at 5 × 10^5^ cells per well in 6-well plates. The following day, the cells were transiently transfected with negative control Hi GC siRNA (10 nM; Thermo Fisher Scientific, Waltham, MA, USA) or BMAL1 siRNA (10, 20, and 50 nM; Integrated DNA Technologies, hs.Ri.ARNTL 13, Coralville, IA) using the Lipofectamine RNAiMAX reagent (Thermo Fisher Scientific, Waltham, WI, MA) according to the manufacturer’s instructions. Cells were used in further experiments 48 h after transfection.

### 4.3. Western blot

Cells were lysed with ice-cold PRO-PREP Protein Extraction Solution (Intron, Daejeon, Korea; 1.0 mM phenylmethylsulfonyl fluoride (PMSF), 1 mM ethylenediaminetetraacetic acid (EDTA), 1 μM pepstatin A, 1 μM leupeptin, and 0.1 μM aprotinin) for 20 min. Lysates were centrifuged at 12,000 rpm for 5 min at 4 °C to remove debris and the supernatants were collected. Lysates (20 μg/well) were separated using 12% sodium dodecyl sulfate polyacrylamide gel electrophoresis, transferred to a polyvinylidene fluoride membrane (Bio-Rad Laboratories, Hercules, CA, USA), and blocked with 5% skim milk in 1× Tris-buffered saline (TBS) for 1 h. The membranes were then incubated with primary antibodies at 4 °C overnight and washed three times with 0.05% Tween-20 in TBS, followed with horseradish peroxidase-conjugated anti-rabbit or anti-mouse secondary antibodies for 1 h. Finally, after the membranes were incubated with enhanced chemiluminescence (ECL) solution (Bio-Rad Laboratories) for 1 min, and signals were detected using an ECL detection system (Bio-Rad Laboratories). The blots were probed with the following primary antibodies: p-AKT (1:1000; Cell Signaling Biotechnologies, Danvers, MA, USA), cyclin B1 (1:1000; Cell Signaling Biotechnologies), cyclin E1 (1:1000; Cell Signaling Biotechnologies), MMP-9 (1:1000; Abcam, Cambridge, UK), BAX (1:1000; Cell Signaling Biotechnologies), BCL-2 (1:1000; Cell Signaling Biotechnologies), caspase-3 (1:1000; Cell Signaling Biotechnologies), BMAL1 (1:2000; Abcam, Cambridge, UK), and β-actin (1:5000; Santa Cruz Biotechnology, Dallas, TX, USA). The band intensity of each protein was quantified with ImageJ software (National Institutes of Health, Bethesda, MD, USA), normalized with β-actin or total-AKT (t-AKT), and then expressed as a percentage of the control (si-NC or Ad-vector).

### 4.4. Immunostaining

Cells were seeded on glass cover slips coated with poly-D-lysine (100 ng/mL; Sigma-Aldrich, St. Louis, MO, USA) at 2.5 × 10^5^ cells per well in 12-well plates. After 24 h, the cells were transfected with siRNAs and incubated for an additional 48 h. The transfected cells were washed three times with 1× phosphate-buffered saline (PBS) and fixed with 4% paraformaldehyde (PFA) in 1× PBS for 10 min. The cells were then permeabilized with 0.3% Triton X-100 in 1× PBS, blocked with 1% bovine serum albumin in 1× PBS for 1 h, incubated overnight at 4 °C with anti-BMAL1 (1:500; Santa Cruz Biotechnology), and exposed to Cy3-conjugated anti-rabbit Immunoglobulin G (IgG) for 1 h. After counterstaining with 4′,6-diamidino-2-phenylindole (DAPI) for 5 min, the coverslips were mounted on glass slides. Images were acquired with a confocal microscope (Leica, Wetzlar, Germany).

### 4.5. 3-(4,5-Dimethylthiazol-2-yl)-2,5-Diphenyltetrazolium Bromide (MTT)Assay

Cells were seeded at 5 × 10^5^ cells/well in 6-well plates. At 24 h post-incubation with siRNAs or adenoviruses, cells were trypsinized, re-plated at 1 × 10^4^ cells per well in 96-well plates, and incubated for an additional 24 h. MTT solution (10 μL of a 5 mg/mL solution) was added to each well and incubated at 37 °C for 2 h. Following this, 200 μL of dimethyl sulfoxide was added and incubated for 30 min. Cell viability was assessed by measuring the absorbance at 590 nm in a microplate reader (Tecan Austria GmbH, Groedig, Salzburg, Austria). The number of cells were calculated as a percentage of that of the control group.

### 4.6. Wound Healing Assay

To monitor the migration of U87MG cells following siRNA or adenoviruses treatment, wound healing assay was carried out with some modification of a previous report [31]. In brief, cells were seeded at 2.5 × 10^5^ cells per well in 12-well plates and incubated with siRNA or adenovirus. At 24 h post-treatment, each well was scratched using a 20μLpipette tip and maintained in minimal medium (Dulbecco’s Modified Eagle Medium (DMEM) supplemented with 2% Fetal Bovine Serum (FBS)) to minimize the cell proliferation for an additional 48 h. The area of wound healing was first measured by ImageJ software using freehand selections mode. The migration of cells toward the wounds was then expressed as percentage of migration: migration (%) = [(At = 0 h—At = 48 h)/At = 0 h] × 100%, where At = 0 h is the area of wound measured immediately after scratching, and At = 48 h is the area of wound measured 48 h after scratching.

### 4.7. Cell Cycle Analysis

To investigate the DNA content in each phase of the cell cycle, flow cytometry was carried out as previously described [29]. Briefly, cells were incubated with siRNA or adenovirus for 48 h, synchronized by serum starvation (0.5% FBS in medium) for 12 h, and allowed to grow in complete medium for 48 h. The cells were then harvested by centrifugation (12,000 rpm, 4 °C, 5 min), washed twice with PBS, and fixed with cold 4% PFA for 10 min. The fixed cells were washed with PBS, stained with propidium iodide (PI; 50 μg/mL) in PBS supplemented with RNase A (100 μg/mL) and 0.05% Triton X-100, and incubated at 37 °C for an additional 30 min. Lastly, the stained cells were filtered through nylon mesh (40 μM) and analyzed by flow cytometry (Becton Dickinson, Franklin Lakes, NJ, USA) to count the cells in each phase of the cell cycle.

### 4.8. Annexin V/propidium Iodide Staining

To distinguish apoptotic cells from living cells, flow cytometry was carried out using the annexin V/propidium iodide (PI) apoptosis kit (Thermo Fisher Scientific) according to the manufacture’s guideline. In brief, U87MG cells (5 × 10^5^ cells per well in 12-well plates) were incubated with adenovirus for 48 h. Then, the cells were trypsinized and treated with annexin V/PI solution for 15 min at room temperature. Finally, we determined the number of apoptotic cells (annexin V/PI-positive) from that of living cells (annexin V-positive) by flow cytometry and represented the percentage of total cells.

### 4.9. Matrigel Invasion Assay

Cells were seeded at 5 × 10^5^ cells per well in 6-well plates. On the next day, cells were incubated with siRNA or adenovirus. At 24 h post-incubation, the cells were harvested by trypsinization, suspended in serum-free medium, and replated on the Matrigel coated-membrane (2 × 10^4^ cells/24-well insert; pore size, 8 mm; Becton Dickinson) using 10% FBS as a chemoattractant. The cells were allowed to move through the pores of the membrane for 24 h, and were stained with 1% cresyl violet solution for visualization. Images were captured using an inverted microscope (Olympus, Tokyo, Japan) and used to count the invading cells.

### 4.10. Adenovirus Production

The open reading frame of the human BMAL1 (ARTNL) gene was first obtained by polymerase chain reaction from a clone (#82189; Addgene, Cambridge, MA) using a primer pair containing EcoRI or XbaI restriction sites (5′-CGA ATT CAT GGC AGA CCA GAG AAT GGA C-3′ and 5′-GTC TAG ACA GCG GCC ATG GCA AGT CAC-3′) and subcloned into the pDonor221-MCS-T2A-mCherry vector to construct the pDonor-BMAL1-T2A-mCherry clone. Then, the final pAd-CMV-BMAL1-T2A-mCheery clone (Ad-BMAL1) was obtained by the LR reaction of Gateway cloning (Thermo Fisher Scientific) between the pDonor-BMAL1-T2A-mCherry and pAd/CMV/V50-DEST vector. Finally, the clone was linearized by digestion with the PacI enzyme and transfected into HEK293A cells grown in a 6-well plate with JetPEI (Polyplus, Illkirch, France) for 2 days. Transfected cells were collected with a cell scraper and lysates were prepared with a brief sonication (30 W, 60 s) to release viruses from the cells. Afterwards, adenoviruses were amplified several times in HEK293A cells in T-75 tissue culture flasks until the titer reached approximately 1 × 10^9^ plaque forming units (pfu)/mL. The viral titer was calculated from the number of serial diluted virus-infected cells exhibiting the mCherry signal under a fluorescence microscope. In this study, U87MG cells were incubated with adenoviruses at 4 × 10^7^ pfu/mL for 2 days, which allowed for stable expression of BMAL1, and then used in further studies. The Ad-CMV-T2A-mCherry (Ad-vector) viruses were used as a negative control. Most U87MG cells (>90%) were infected with Ad-vector or Ad-BMAL1 viruses as determined by red fluorescence under a fluorescent microscope.

### 4.11. Statistical Analysis

The data are expressed as the mean ± standard error of the mean (SEM). The statistical significance of differences between two groups or multiple groups was compared by unpaired Student’s *t*-test or one-way analysis of variance (ANOVA) followed by an appropriate multiple comparison test. *p*-values <0.05 were considered statistically significant. All statistical analyses were performed using GraphPad Prism 6 (GraphPad Software Inc., La Jolla, CA, USA).

## 5. Conclusions

In summary, our results demonstrated that BMAL1 as an anti-glioblastoma gene suppresses proliferation, migration, and invasion of U87MG cells by the downregulation of cyclin B1, p-AKT, and MMP-9. Therefore, BMAL1 may be a potential therapeutic target in the treatment of glioblastoma.

## References

[1] Gallego O. (2015). Nonsurgical treatment of recurrent glioblastoma. Curr. Oncol..

[2] Kim T.-G., Kim C.-H., Park J.-S., Park S.-D., Kim C.K., Chung D.-S., Hong Y.-K. (2010). Immunological factors relating to the antitumor effect of temozolomide chemoimmunotherapy in a murine glioma model. Clin. Vaccine Immunol..

[3] Semenza G.L. (2008). A new weapon for attacking tumor blood vessels. N. Engl. J. Med..

[4] Jacinto F.V., Esteller M. (2007). MGMT hypermethylation: A prognostic foe, a predictive friend. DNA Repair.

[5] Edgar R.S., Green E.W., Zhao Y., Van Ooijen G., Olmedo M., Qin X., Xu Y., Pan M., Valekunja U.K., Feeney K.A. (2012). Peroxiredoxins are conserved markers of circadian rhythms. Nature.

[6] Ye R., Selby C.P., Ozturk N., Annayev Y., Sancar A. (2011). Biochemical analysis of the canonical model for the mammalian circadian clock. J. Biol. Chem..

[7] Savvidis C., Koutsilieris M. (2012). Circadian rhythm disruption in cancer biology. Mol. Med..

[8] Kurose T., Yabe D., Inagaki N. (2011). Circadian rhythms and diabetes. J. Diabetes Invest..

[9] Boyce P., Barriball E. (2010). Circadian rhythms and depression. Aust. Fam. Physician.

[10] He Q., Wu B., Price J.L., Zhao Z. (2017). Circadian rhythm neuropeptides in Drosophila: Signals for normal circadian function and circadian neurodegenerative disease. Int. J. Mol. Med..

[11] Duffy J.F., Zitting K.-M., Chinoy E.D. (2015). Aging and circadian rhythms. Sleep Med. Rev..

[12] Wilking M., Ndiaye M., Mukhtar H., Ahmad N. (2013). Circadian rhythm connections to oxidative stress: Implications for human health. Antioxid.Redox Signal..

[13] Saha S., Sassone-Corsi P. (2007). Circadian clock and breast cancer: A molecular link. Cell Cycle.

[14] Winter S.L., Bosnoyan-Collins L., Pinnaduwage D., Andrulis I.L. (2007). Expression of the circadian clock genes Per1 and Per2 in sporadic and familial breast tumors. Neoplasia (New York, NY).

[15] Cao Q., Gery S., Dashti A., Yin D., Zhou Y., Gu J., Koeffler H.P. (2009). A role for the clock gene per1 in prostate cancer. Cancer Res..

[16] Tokunaga H., Takebayashi Y., Utsunomiya H., Akahira J.-I., Higashimoto M., Mashiko M., Ito K., Niikura H., Takenoshita S.-I., Yaegashi N. (2008). Clinicopathological significance of circadian rhythm-related gene expression levels in patients with epithelial ovarian cancer. Acta.Obstet. Gynecol. Scand..

[17] Taniguchi H., Fernández A.F., Setién F., Ropero S., Ballestar E., Villanueva A., Yamamoto H., Imai K., Shinomura Y., Esteller M. (2009). Epigenetic inactivation of the circadian clock gene BMAL1 in hematologic malignancies. Cancer Res..

[18] Jung-Hynes B., Huang W., Reiter R.J., Ahmad N. (2010). Melatonin resynchronizes dysregulated circadian rhythm circuitry in human prostate cancer cells. J. Pineal Res..

[19] Sakamoto W., Takenoshita S. (2015). Overexpression of both clock and Bmal1 inhibits entry to S phase in human colon cancer cells. Fukushima J. Med. Sci..

[20] Jung C.-H., Kim E.M., Park J.K., Hwang S.-G., Moon S.-K., Kim W.-J., Um H.-D. (2013). Bmal1 suppresses cancer cell invasion by blocking the phosphoinositide 3-kinase-Akt-MMP-2 signaling pathway. Oncol. Rep..

[21] Elshazley M., Sato M., Hase T., Yamashita R., Yoshida K., Toyokuni S., Ishiguro F., Osada H., Sekido Y., Yokoi K. (2012). The circadian clock gene BMAL1 is a novel therapeutic target for malignant pleural mesothelioma. Int. J. Cancer.

[22] Elmore S. (2007). Apoptosis: A review of programmed cell death. Toxicol. Pathol..

[23] Zeng Z.-l., Luo H.-y., Yang J., Wu W.-j., Chen D.-l., Huang P., Xu R.-h. (2014). Overexpression of the circadian clock gene Bmal1 increases sensitivity to oxaliplatin in colorectal cancer. Clin. Cancer Res..

[24] Tang Q., Cheng B., Xie M., Chen Y., Zhao J., Zhou X., Chen L. (2017). Circadian clock gene Bmal1 inhibits tumorigenesis and increases paclitaxel sensitivity in tongue squamous cell carcinoma. Cancer Res..

[25] Jiang W., Zhao S., Jiang X., Zhang E., Hu G., Hu B., Zheng P., Xiao J., Lu Z., Lu Y. (2016). The circadian clock gene Bmal1 acts as a potential anti-oncogene in pancreatic cancer by activating the p53 tumor suppressor pathway. Cancer Lett..

[26] Papagiannakopoulos T., Bauer M.R., Davidson S.M., Heimann M., Subbaraj L., Bhutkar A., Bartlebaugh J., Vander Heiden M.G., Jacks T. (2016). Circadian rhythm disruption promotes lung tumorigenesis. Cell Metab..

[27] Li S., Szymborski A., Miron M., Marcellus R., Binda O., Lavoie J., Branton P. (2009). The adenovirus E4orf4 protein induces growth arrest and mitotic catastrophe in H1299 human lung carcinoma cells. Oncogene.

[28] Shen H., Hau E., Joshi S., Dilda P.J., McDonald K.L. (2015). Sensitization of glioblastoma cells to irradiation by modulating the glucose metabolism. Mol. Cancer Ther..

[29] Kiessling S., Beaulieu-Laroche L., Blum I.D., Landgraf D., Welsh D.K., Storch K.-F., Labrecque N., Cermakian N. (2017). Enhancing circadian clock function in cancer cells inhibits tumor growth. BMC Boil..

[30] Rogakou E.P., Nieves-Neira W., Boon C., Pommier Y., Bonner W.M. (2000). Initiation of DNA fragmentation during apoptosis induces phosphorylation of H2AX histone at serine 139. J. Biol.Chem..

[31] Yue P.Y., Leung E.P., Mak N., Wong R.N. (2010). A simplified method for quantifying cell migration/wound healing in 96-well plates. J. Biomol. Screen..

